# Wild bird trade at live poultry markets potentiates risks of avian influenza virus introductions in Iran

**DOI:** 10.1080/20008686.2021.1992083

**Published:** 2021-11-08

**Authors:** Amir Modirihamedan, Shabnam Aghajantabar, Jacqueline King, Annika Graaf, Anne Pohlmann, Leila Aghaiyan, Zahra Ziafati Kafi, Yeganeh Mahfoozi, Hossein Hosseini, Martin Beer, Arash Ghalyanchilangeroudi, Timm Harder

**Affiliations:** a Department of Microbiology and Immunology, Faculty of Veterinary Medicine, University of Tehran, Tehran, Iran; b Institute of Diagnostic Virology, Friedrich-Loeffler-Institut, Germany; c Department of Avian Medicine, School of Veterinary Medicine, Shiraz University, Shiraz, Iran; d Department of Clinical Sciences, Faculty of Veterinary Medicine, Islamic Azad University, Karaj Branch, Karaj, Iran

**Keywords:** Avian influenza, wild birds, poultry, live bird market, Zoonosis, Iran

## Abstract

Wild aquatic birds are the main natural host reservoir of avian influenza viruses (AIV). Migratory aquatic birds can translocate AI viruses over wide geographic distances. AIV may be transmitted reciprocally at the wild bird–poultry interface, increasing viral variability and potentially driving the zoonotic potential of these viruses. A cross-sectional study on AIV and several further avian viral pathogens conducted in 396 trapped migratory aquatic birds traded at live bird markets (LBM) in northern Iran identified 11 AIV-positive cases. The 10 identified H9N2 viral sequences fell into wild bird H9 lineage Y439; in addition, an H10N3 virus of Eurasian lineage was detected. Ten samples contained low viral loads of avian coronavirus but could not be further characterized. Although traditional trading of live-trapped wild birds provides income for hunters, particularly during fall migration periods, it increases the risk of introducing new AIV strains from the natural reservoir to poultry kept at LBMs and, potentially, to traders and customers. Banning these birds from poultry trading lines would lower such risks considerably.

## Introduction

Live bird markets (LBMs) provide freshly slaughtered poultry meat, thus essential for providing high-quality animal protein to rural and urban populations in Iran as well as in many further Middle East, Asian, and African countries [1]. Studies have shown that LBMs can act as a hub for various avian pathogens, including the avian influenza viruses (AIV) [2,3]. Wild aquatic birds are the largest natural reservoir for AIV, maintaining a high degree of viral diversity of at least 16 hemagglutinin (HA) and nine neuraminidase (NA) subtypes [4]. Virus transmission in this reservoir is achieved mainly via the fecal-oral route. Virus replication usually remains localized to the respiratory and gastrointestinal epithelia and does not cause overt clinical signs in the individual infected bird. Such viruses that are perpetuated in the natural host reservoir are of low pathogenicity, LP [5]. Rapid annual turnover rates of wild waterbird metapopulations and mixing and mingling of different species of various geographic origins during migratory periods provide ideal conditions for the efficient spread of AIV within the natural reservoir and increase viral diversity by reassortment [5].

Introduction of LPAIV to poultry may result in the establishment of endemic infection cycles in susceptible poultry populations. In the case of subtypes H5 and H7, replication in gallinaceous poultry is associated with a risk of a spontaneous mutation affecting the sequence encoding the endoproteolytic cleavage site of the hemagglutinin (HA) protein. This causes a shift towards a high pathogenicity (HP) phenotype, and such variants are capable of systemic infection correlated with high mortality [6,7]. The spillback of such HPAIV from poultry into wild bird populations may lead to the mobilization of these viruses with subsequent spread along migratory flyways. Such an event took place in geese in Guangdong province (Southern China) in 1996 and generated an HPAIV of subtype H5N1. Since then, HPAIV of the goose/Guangdong (gs/GD) lineage established endemic infections in poultry populations of several Southeast Asian and South Asian countries and in the Near and Middle Eastern regions. Trading connections and spread with migratory wild birds continue to invoke epidemic waves of gs/GD HPAIV descendants in European, African, and North American countries [8].

Other AIV subtypes can be introduced to and become entrenched in poultry populations as well. Endemic infections of H9N2 AIV are reported from commercial poultry of Asian, Middle Eastern, and North African countries [9]. H9N2 viruses have been classified into Eurasian and American lineages; the Eurasian lineage further diversified into G1, Y439 (aka Eurasian wild bird lineage), Y280, and F98 clusters, some of which bear viruses with a zoonotic potential [10–12]. Although all H9N2 AIVs have been categorized as LPAI, they can invoke significant economic losses in commercial poultry production [13]. In addition, due to their widespread occurrence, H9N2 viruses are important donors of genome segments in reassortment events including HPAIV of the gs/GD lineage [14].

Iran is located at the crossing of important migratory flyways such as the Central Asian, East Asian-East Africa, and the Black Sea-Mediterranean flyway. The presence of various AIV sub- and pathotypes has repeatedly been documented in migrating wild birds and poultry in Iran [15]. Iran has a strong poultry industry, and poultry trading via live bird markets is regionally important [16]. Therefore, the country potentially can be a hotspot of AIV transmission [17].

This study primarily focused on AIV infections in wild birds traded in live bird markets in the northern provinces of Iran. It was hypothesized that wild migratory aquatic birds might play a critical role in introducing AIV to live bird markets.

## Material and methods

### Sample origin

On different days during October 2019, swabs were taken from 396 individuals from four different species of wild migratory aquatic birds, all traded at different LBMs of Ferydunkenar city, Mazandaran province, Iran. The province borders the south coast of the Caspian Sea and provides resting and/or wintering sites during fall and winter for migratory waterbirds along several migratory flyways [18]. Swab samples were collected from mallards (*Anas platyrhynchos*, n = 96 cloacal swabs), Eurasian teal (*Anas crecca*, n = 100 cloacal swabs), Eurasian coots (*Fulica atra*, n = 100, comprising 40 oropharyngeal and 60 cloacal swabs), and greater white-fronted geese (*Anser albifrons*, n = 100 cloacal swabs). Due to the limited compliance of the owners of the birds, a full set of cloacal and oropharyngeal swabs could not be obtained. A swab smear with 125 µL of transport medium was applied onto an FTA card spot (Kawsar DNA Banking cards, Kawsar, Iran) to preserve nucleic acid integrity and inactivate viral infectivity. FTA cards were shipped to the National Reference Laboratory for Avian Influenza (NRL-AI), Friedrich-Loeffler-Institut (FLI), Germany. At the time of sample collection, neither outbreaks of notifiable AI nor notifiable Newcastle Disease (ND) in poultry or wild birds had been reported from these regions.

### Detection of avian influenza virus by PCR

Nucleic acid was extracted from FTA card spots of each sample (oropharyngeal or cloacal) by using a Nucleomag® Vet kit (Macherey-Nagel, Düren, Germany) in a BioSprint 96 device (Qiagen, Hilden, Germany). Samples were tested for AIV-specific RNA using an internally controlled Taq-man real-time reverse transcriptase PCR (RT-qPCR) detecting a fragment of the matrix (M) gene [19]. AIV-positive samples were subtyped by RT-qPCR using the Riems Influenza A Subtyping Assay (RITA) [20]. Samples with virus loads of Cq ≤ 28 were subjected to full genome next-generation sequencing. Other positive samples were Sanger–sequenced. In brief, HA and NA genes of those samples were amplified by conventional RT-PCR using overlapping sets of amplificates (primer sequences for both H9 and H10 in Supplemental Table 1).

**Table 1.** Iranian wild bird samples obtained in October 2019 from several live bird markets in northern Iran and testing positive for avian influenza virus

Bird species	Swab sample	Subtype	Cq	Accession number
Eurasian teal	Cloacal-137	H9N2	22.78	MZ277345 (HA)- MZ277346 (MP) – MZ277347 (NA) – MZ277348 (NP) – MZ277349 (NS) – MZ277350 (PA) – MZ277351 (PB1)- MZ277352 (PB2)
Eurasian teal	Cloacal-140	H9N2	24.05	MZ277330 (HA)
Eurasian teal	Cloacal-148	H9N2	26.00	MZ277331 (HA)
Eurasian teal	Cloacal-158	H9N2	21.71	MZ277332 (HA)
Eurasian teal	Cloacal-163	H9N2	23.76	MZ277353 (HA)- MZ277354 (MP)- MZ277355 (NA) – MZ277356 (NP) – MZ277357 (NS) – MZ277358 (PA) – MZ277359 (PB1) -MZ277360 (PB2)
Eurasian teal	Cloacal-166	H9N2	24.31	MZ277333 (HA)
Eurasian teal	Cloacal-167	H9N2	27.62	MZ277334 (HA)
White-fronted goose	Cloacal-238	H9N2	29.36	MZ277337 (HA)- MZ277338 (MP) – MZ277339 (NA)- MZ277340 (NP)- MZ277341 (NS)- MZ277342 (PA) – MZ277343 (PB1) -MZ277344 (PB2)
White-fronted goose	Cloacal-239	H9N2	26.66	MZ277361 (HA) – MZ277368 (MP)- MZ277361 (NA) – MZ277362 (NP)- MZ277363 (NS) – MZ277364 (PA)- MZ277365 (PB1) -MZ277366 (PB2)
White-fronted goose	Cloacal-268	H9N2	21.57	MZ277369 (HA)- MZ277370 (MP)- MZ277371 (NA) – MZ277372 (NP) – MZ277373 (NS) – MZ277374 (PA) – MZ277375 (PB1) -MZ277376 (PB2)
White-fronted goose	Cloacal-298	H10N3	30.82	MZ277336(HA), MZ562475 (NA)

Cq – RT-qPCR on generic M-gene target; indirect measure of viral RNA load.

### Detection of other avian viral pathogens

RT-qPCRs were used to examine avian metapneumoviruses, avian herpesvirus, avian parvovirus, avian bornavirus, and avian parvovirus as described elsewhere [21].

### Sequencing

Five RNA samples extracted from cloacal samples on FTA cards and positive for H9N2 were sequenced on the Mk1C MinION platform (Oxford Nanopore Technologies – ONT, Oxford, UK) after universal amplification, as previously described [22]. In short, the extracted RNA was amplified with an influenza-specific primer pair designed to bind to the conserved end regions of all segments and Invitrogen Superscript III One-Step RT-PCR Kit with Platinum Taq (Thermo Fisher Scientific, Waltham, USA). The PCR products were purified with AMPure XP Magnetic Beads in a 0.65× sample volume to bead volume ratio (Beckman Coulter, Fullerton, USA). Whole-genome sequencing of the purified PCR amplicons was conducted on the Mk1C MinION platform (ONT) utilizing the transposase-based Rapid Barcoding Kit (RBK-004, ONT) according to the manufacturer’s instructions. After library preparation and pooling, the barcoded and adapter-ligated samples were loaded onto an FLO-MIN106 R9.4.1 flow cell (ONT). A four-hour run with the MinKNOW software (v20.06.15, ONT) and real-time base caller Guppy (v.4.0.11, ONT) in the setting ‘fast basecalling’ produced demultiplexed, quality checked, and trimmed raw data. For consensus production, sequencing data was assembled with the Geneious Prime software (v.2021.0.1, Biomatters, Auckland, New Zealand) in a map-to-reference approach with MiniMap2. Representative sequences for different lineages of both H9 and H10 strains were obtained from GenBank. All sequences were deposited in the NCBI database (Table 1).

The HA and neuraminidase (NA) gene segments of a further five H9N2 positive samples and another sample testing positive for H10N3 were Sanger-sequenced according to previously published methods [21]. Primers used for Sanger sequencing are shown in Supplemental Table 1.

### Phylogenetic analyses

Alignment and identity matrices were established using Geneious or MAFFT programs [23]. The maximum likelihood phylogenetic analysis was carried out using the IQ Tree software, version 2.1.1 [24]. ModelFinder [25] included in to the IQ Tree software suite was used to select the best fitting codon-based model according to the Bayesian informative criterion. The HA phylogenetic tree was edited, designed, and viewed using the FigTree v1.4.4 software (http://tree.bio.ed.ac.uk/software/figtree/) and Inkscape 0.92.

## Results

**Figure 1.**
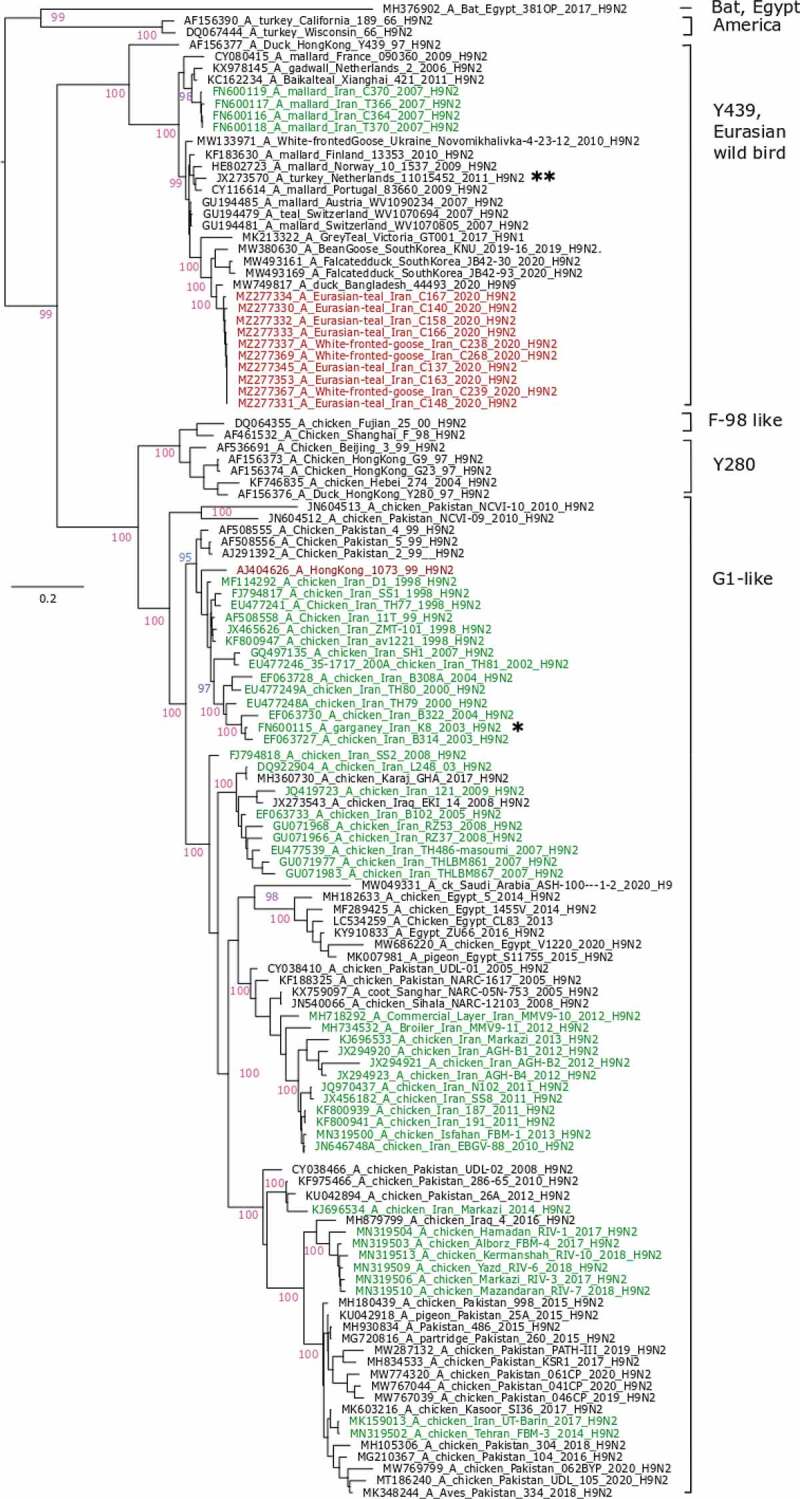
Phylogenetic analysis of the hemagglutinin open reading frame of H9N2 viruses from Iran. Trees were generated by maximum likelihood calculations using the IQ Tree software version 2.1.1 applying the best fit codon-based model according to the Bayesian informative criterion MG+F1X4+G4. Numbers at nodes signal robustness according to ultrafast bootstrap support. Red colored sequences have been established in this study. Other sequences from Iran have been labelled green. Clades within the H9 HA subtype are indicated to the right of the tree. Asterisks denote switches of Y439- and G1-like viruses between wild bird and poultry populations as explained in the discussion

In this study, 3.1% of cloacal samples (11 out of 356) were detected as influenza A virus positive with Cq values ranging from 21 to 35 (Table 1). None of the forty oropharyngeal samples obtained from coots tested positive for AIV RNA. The frequency of AIV detection in cloacal samples was 7% in Eurasian teals and 4% in greater white-fronted geese. AIV was not detected in samples of Eurasian coots and mallards. Ten out of eleven positive samples were subtyped as H9N2, while the remaining sample was identified as H10N3. Five positive H9N2 with Cq values <28 yielded full genome sequences. The obtained sequences showed a very high homology among them. HA and NA genes of all other five H9N2-positive samples were Sanger-sequenced. Phylogenetic analyses revealed that H9 HA and N2 NA genes clustered within the Y439, aka Eurasian wild bird, lineage (HA open reading frame: Figure 1; NA: Supplemental Figure 1(e)). All internal genes (PB2, PB1, PA, NP, M, and NS) of the five fully sequenced H9N2 viruses were analyzed in BLAST searches against the NCBI GenBank database. The PB1 and PB2, respectively, had highest identity to A/duck/Bangladesh/33137/2017 (H3N2) (97.9%) and A/duck/Bangladesh/30828/2016 (H3N8) (97.5%). The PA gene was closely related to A/chicken/Bulgaria/77_20VIR1727/2020 (H5N2) (98.3). NP, MP, and NS genes, respectively, were related to A/garganey/North_Kazakhstan/45/2018 (H3N8) (99.2), A/duck/Mongolia/961/2019 (H3N8) (99.4%), and A/domestic-duck/Georgia/4/2016 (H4N6) (98.9). Phylogenetic analyses confirmed that all internal segments are of Eurasian origin, and no reassortment occurred within the set of the five fully sequenced H9N2 viruses (Supplemental Figure 1(a–i)). No mutations that would signal antiviral resistance or adaptation to mammalian/human host species were identified in these sequences according to the FluSurver website (https://flusurver.bii.a-star.edu.sg/).

For the H10N3-positive sample, only the HA and NA ORFs were analyzed due to the comparatively low viral load (Table 1). Both the HA H10 and NA N3 genes segregated into Eurasian lineages (Supplemental Figure 1(j–k)).

Ten additional samples reacted positive for avian coronaviruses; however, due to low viral loads, could not be further characterized. None of the other avian viral pathogens investigated here were detected in our samples.

## Discussion

Here, we report the detection of 10 H9N2 of the Y439 lineage and one H10N3-positive sample in aquatic wild birds traded at LBMs in northern Iran. The prevalence of active AIV infections detected here is similar to that described in a previous survey (2003 to 2007) in Iran, where 3% of wild bird samples were AIV positive by RT-PCR, including H9N2. In the respective study, all except one H9N2 virus for which HA gene sequences were available also fell into the Y439 lineage. However, a single H9N2 virus from a garganey (*Spatula querquedula*) clustered in the G1 lineage is closely related to contemporary poultry H9N2 viruses from Iran ([26]; Figure 1, marked by an asterisk). This indicated transmissibility of chicken-adapted G1 H9N2 viruses to aquatic wild birds. Although Y439-like H9N2 viruses have not been detected in poultry in Iran so far, such viruses have been described in turkeys in the Netherlands, Poland, and the UK [27]. Thus, H9N2 viruses of different lineages appear to be transmissible across the wild bird/poultry interface. Similar transmission patterns across interfaces are also evident for HPAIV of the gs/GD lineage in Iran [17].

The history of H9N2 infection in Iranian poultry is dominated by incursions and circulation of descendants of the G1 lineage, subsequently forming several distinguishable clusters (Figure 1, green colored sequences). For several of these clusters an immediate ancestor can be found in one of the neighboring countries of Iran, Iraq, or Pakistan. This suggests incursions of G1-like H9N2 to Iran by transboundary poultry trade rather than by wild birds [28,29]. Unlike the Y439 wild bird H9N2 viruses examined here, the Iranian G1-like viruses harbor mutations (e.g., HA Q226L) that increase their zoonotic potential [28–30]. In addition, sets of internal genes of H9N2 viruses have been widely implicated in reassortment events with HPAIV of the gs/GD lineage and with other subtypes, including H10 [10–12].

The H10N3 virus was detected here in a white-fronted goose sample and clustered with other Eurasian wild bird viruses (Supplemental Figure 1(j–k)). Eurasian H10 viruses have been detected in a wide geographical range [31]. Similar to subtype H9, some H10 viruses have been found to express zoonotic potential and have sporadically infected humans (in China and Australia) [32,33] and possibly other mammalian species [34].

The impact of LBMs as a hub of zoonotic avian pathogens, in particular influenza viruses, has been widely demonstrated [31]. Applying restriction measures to LBMs, including temporal closures, slowed down and even interrupted the circulation of these viruses and, hence, reduced the risk of human exposure [35,36]. Surveillance studies on influenza at Iranian LBMs are scarce, although one study [37] indicated H9N2 seroprevalence rates of 26.3% and active infections of 9.2%, while in another study 53% of samples were seropositive for H9 [38]. High incidences of H9N2 infections in LBM poultry are also reported from neighboring Pakistan [39]. In northern parts of Iran, poultry owners and/or traders stock local LBMs, and hunters provide aquatic wild birds that are often captured alive and slaughtered on-demand at LBM. Such practices violate previously evaluated measures aiming to limit AIV circulation and reassortment at LBMs. These LBMs, in contrast, provide a favorable environment for enhanced mixing of AIV of wild bird and poultry origin while exposing human hosts to such viruses. LBMs remain indispensable in several regions where poultry abattoirs and sustained cold chains are missing. Yet, low-level biosecurity detected especially at rural LBMs is difficult to improve where poor socio-economic status and educational background conflicts with the advancement of hygiene precautions [40–42].

The infectiological conflicts arising from LBMs and wet markets in general and from LBMs trading wild birds in parallel are evident from the literature and are exemplified here for the situation in northern Iran. The authoritarian closure of such markets unlikely provides a sustainable solution since traders and buyers depend, at least regionally, on the income and offer of poultry meat, respectively. Thus, the risks of establishing black markets following LBM closure are high. Assuming that hunting aquatic wild birds in northern Iran is indispensable to provide income for hunters and their families, the prohibition of hunting likewise will miss the mark. Although there is no simple solution at hand, establishing separate routes for marketing wild birds and poultry clearly has a high priority.

## Supplementary Material

Supplemental Material

## Data Availability

All data established are either shown in the manuscript or are available at public databases (NCBI GenBank). https://www.ncbi.nlm.nih.gov/nucleotide/.
