# Supplementary material for: Wild bird trade at live poultry markets potentiates risks of avian influenza virus introductions in Iran
Source: Infect Ecol Epidemiol. 2021 Nov 8;11(1):1992083. doi: 10.1080/20008686.2021.1992083 (PMC8583743; doi:10.1080/20008686.2021.1992083)
Supplement: Supplemental Material [file ZIEE_A_1992083_SM9565.docx]

**Supplemental figure 1. .** Phylogenetic analysis of the open reading frames of H9N2 (A-I) and H10N3 (J-K) viruses from Iran. Trees were generated by maximum likelihood calculations using the IQ Tree software version 2.1.1 applying the best fit codon based model according to the Bayesian informative criterion. Numbers at nodes signal robustness according to ultrafast bootstrap support. Red colored sequences have been established in this study. Other sequences from Iran have been labelled green.


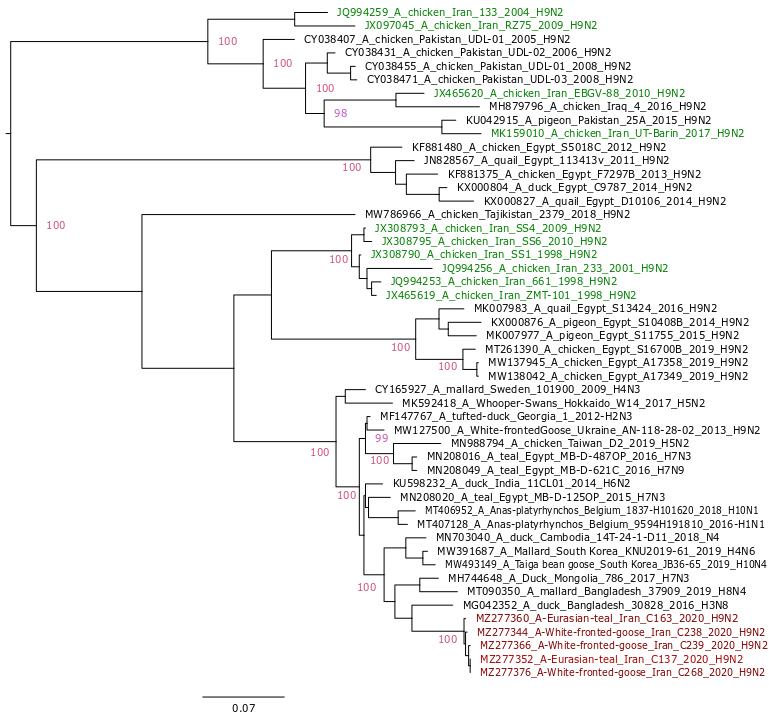


Supplemental figure 1A. PB2


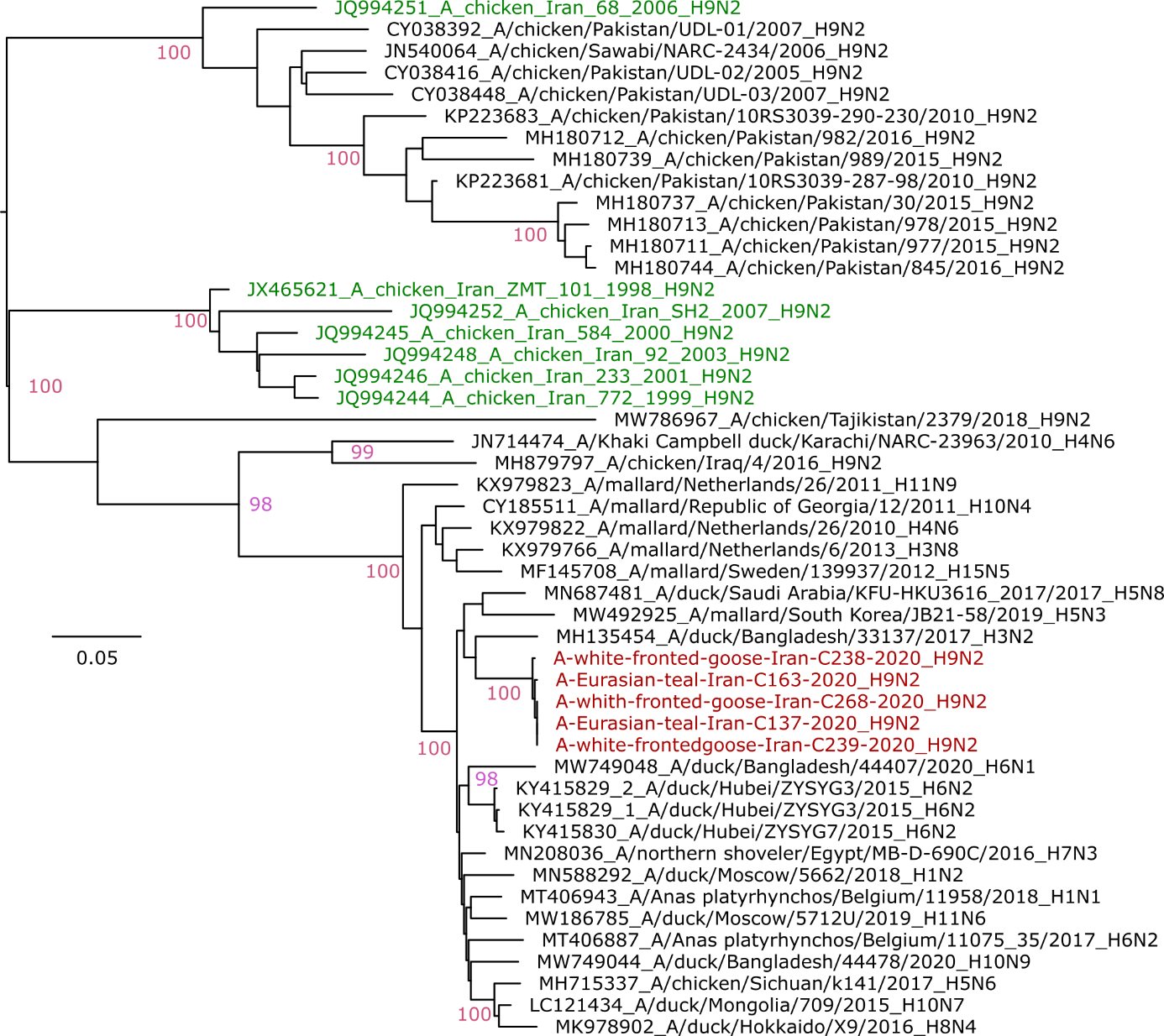


Supplemental figure 1B. PB1


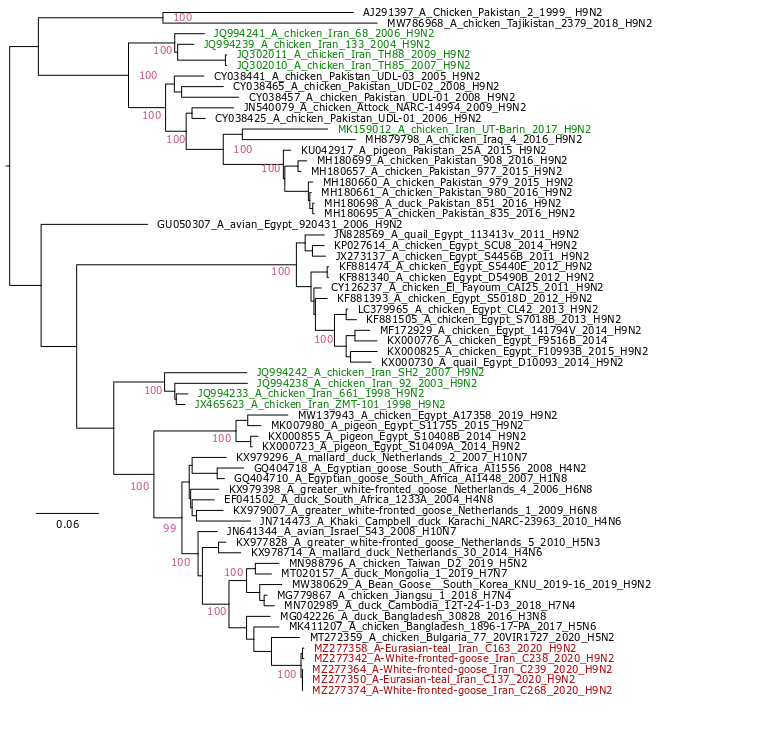


Supplemental figure 1C. PA


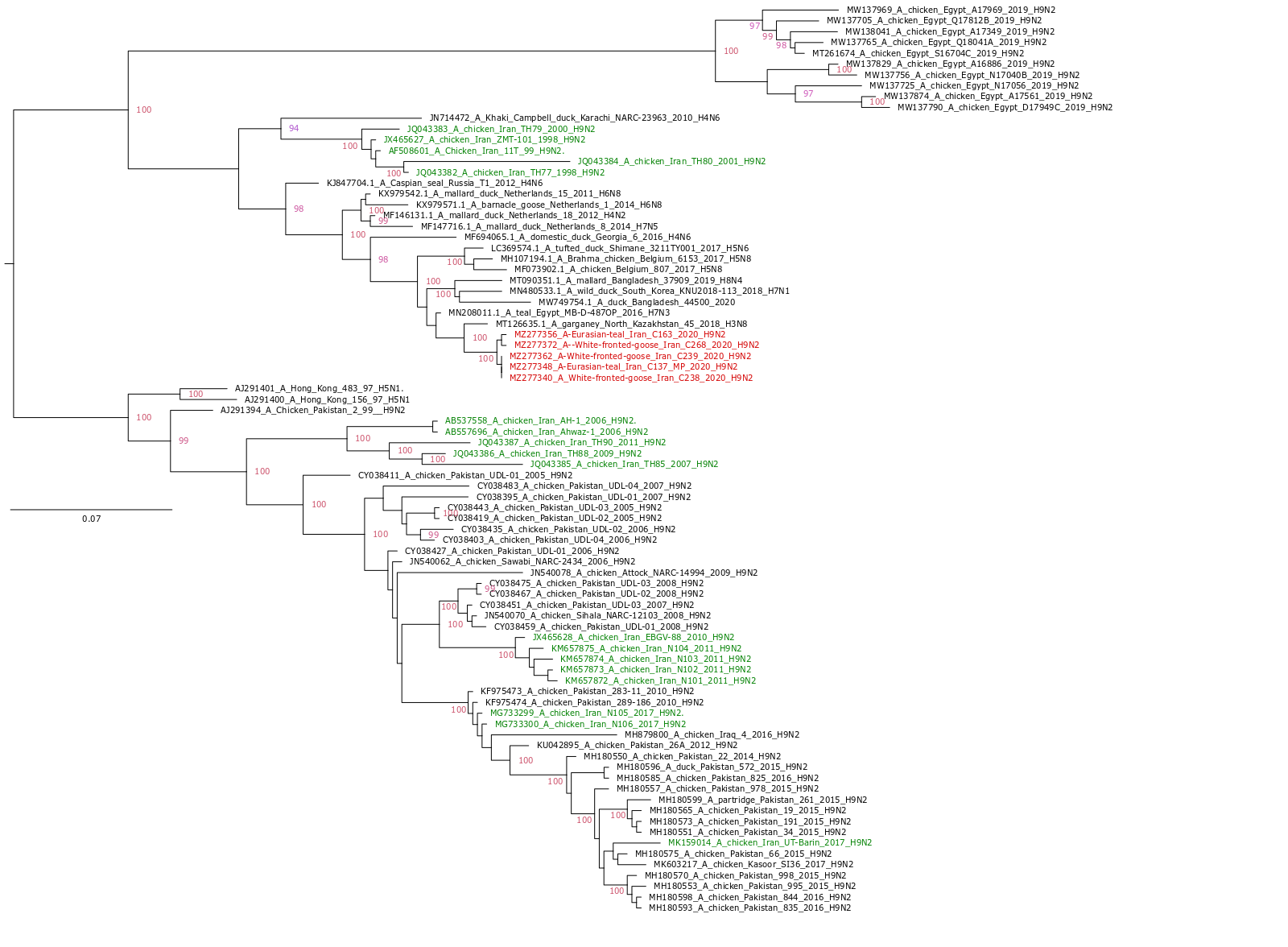


Supplemental figure 1D. NP


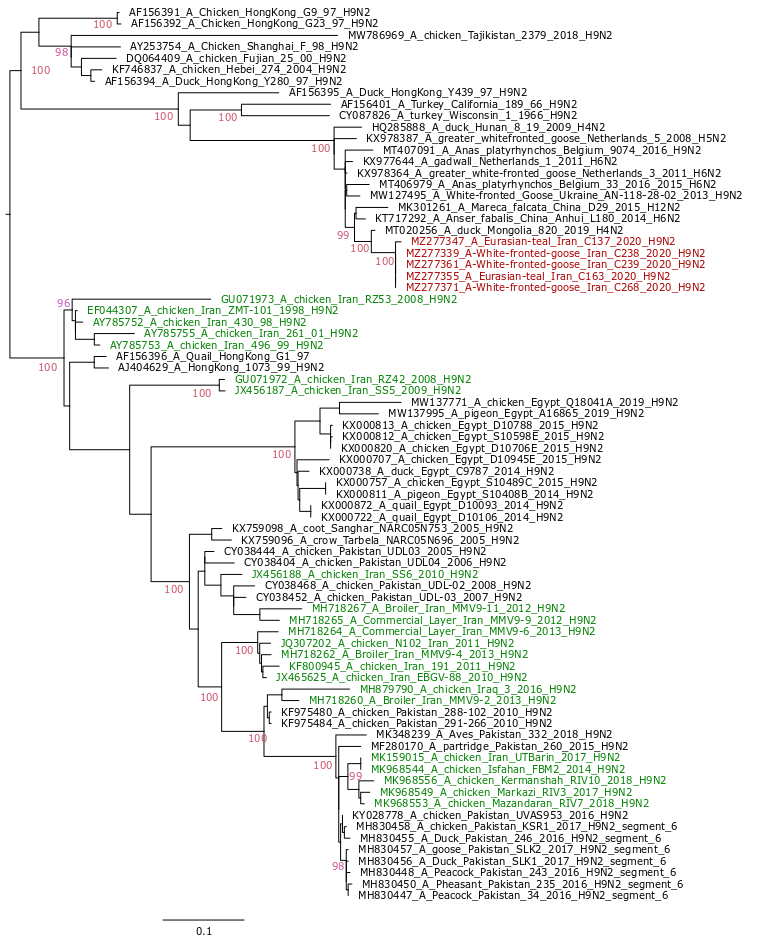


Supplemental figure 1E. NA, subtype N2


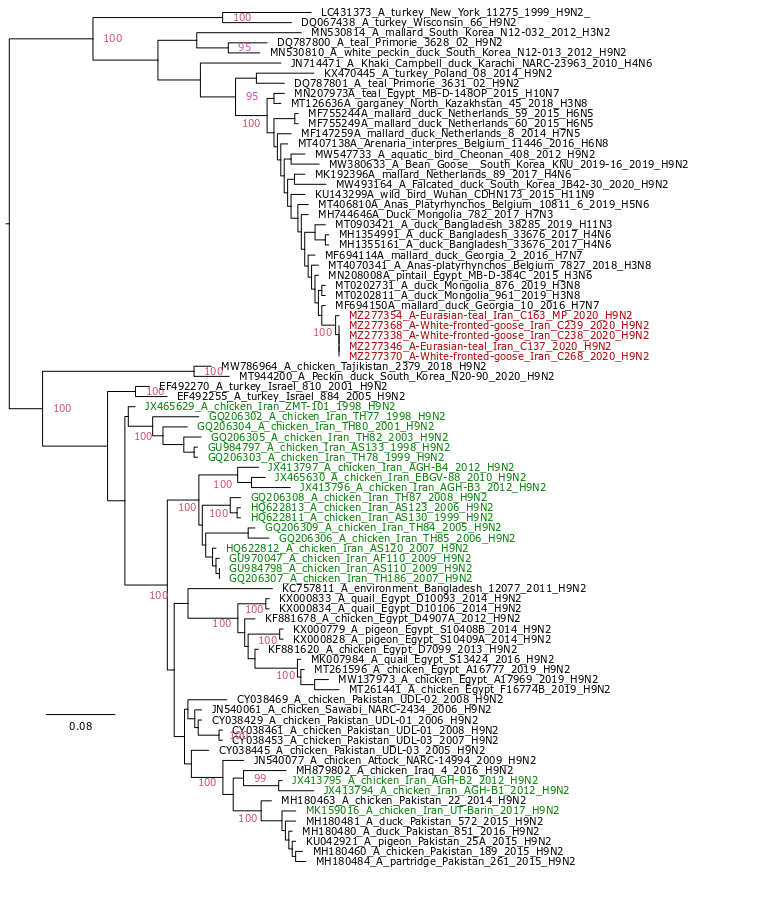


Supplemental figure 1F. M1


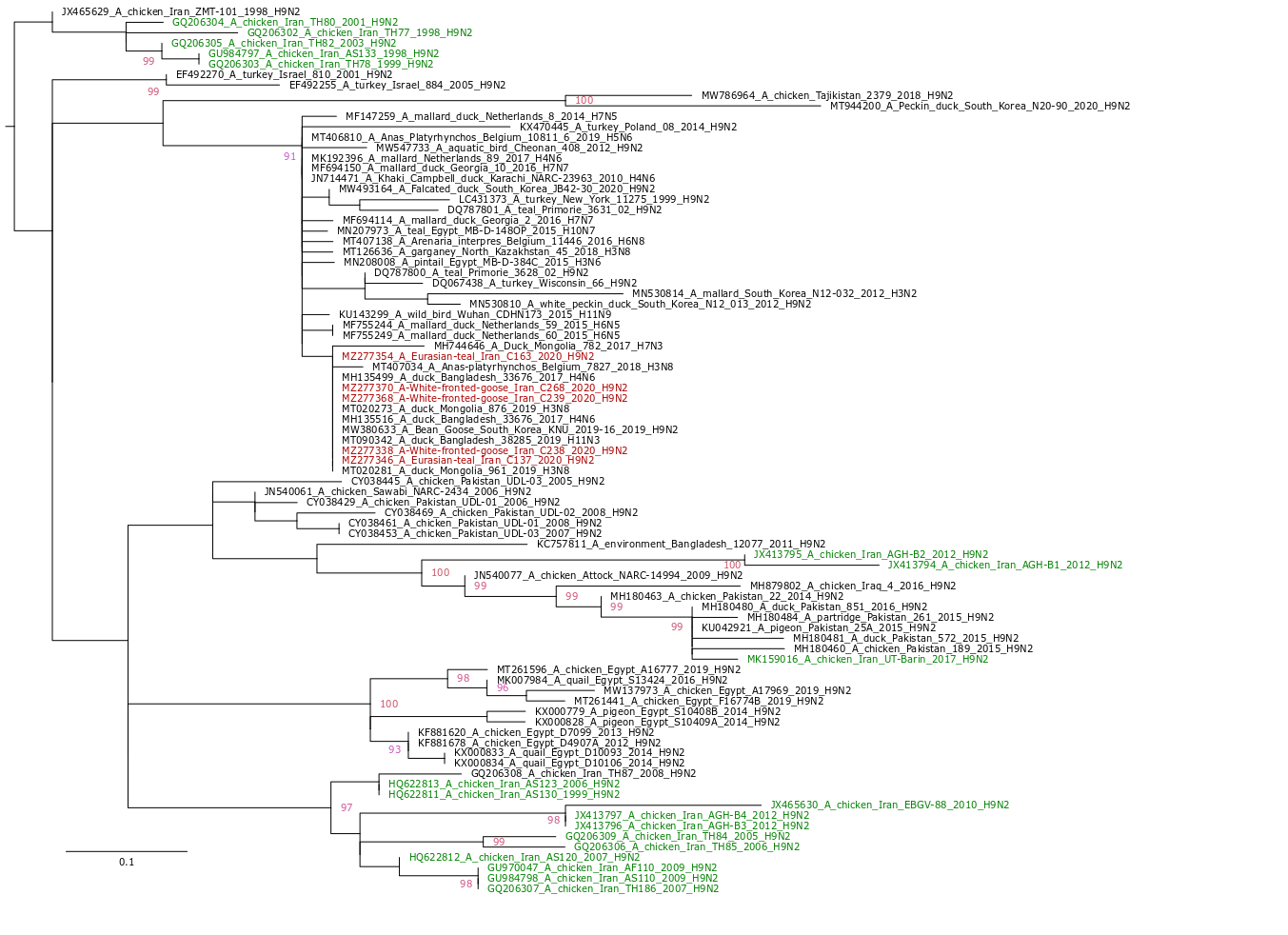


Supplemental figure 1G. M2


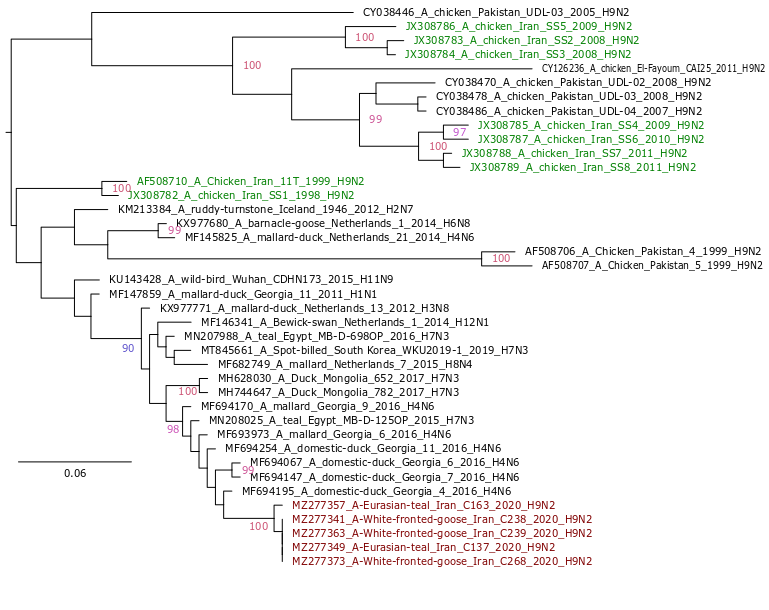


Supplemental figure 1H. NS1


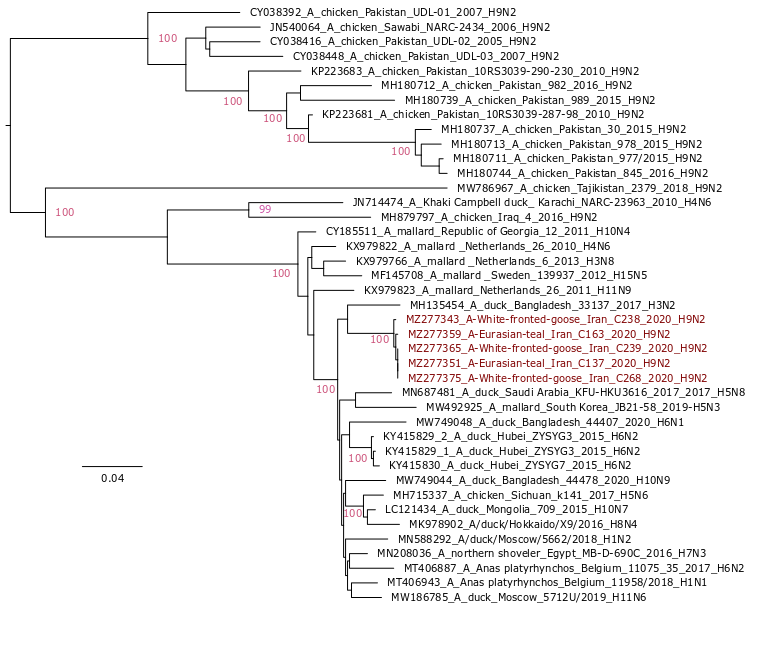


Supplemental figure 1I. NS2


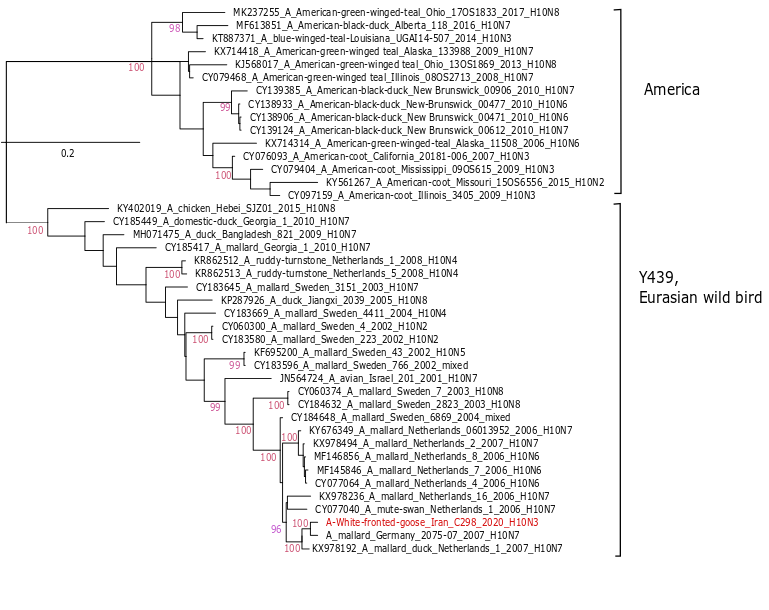


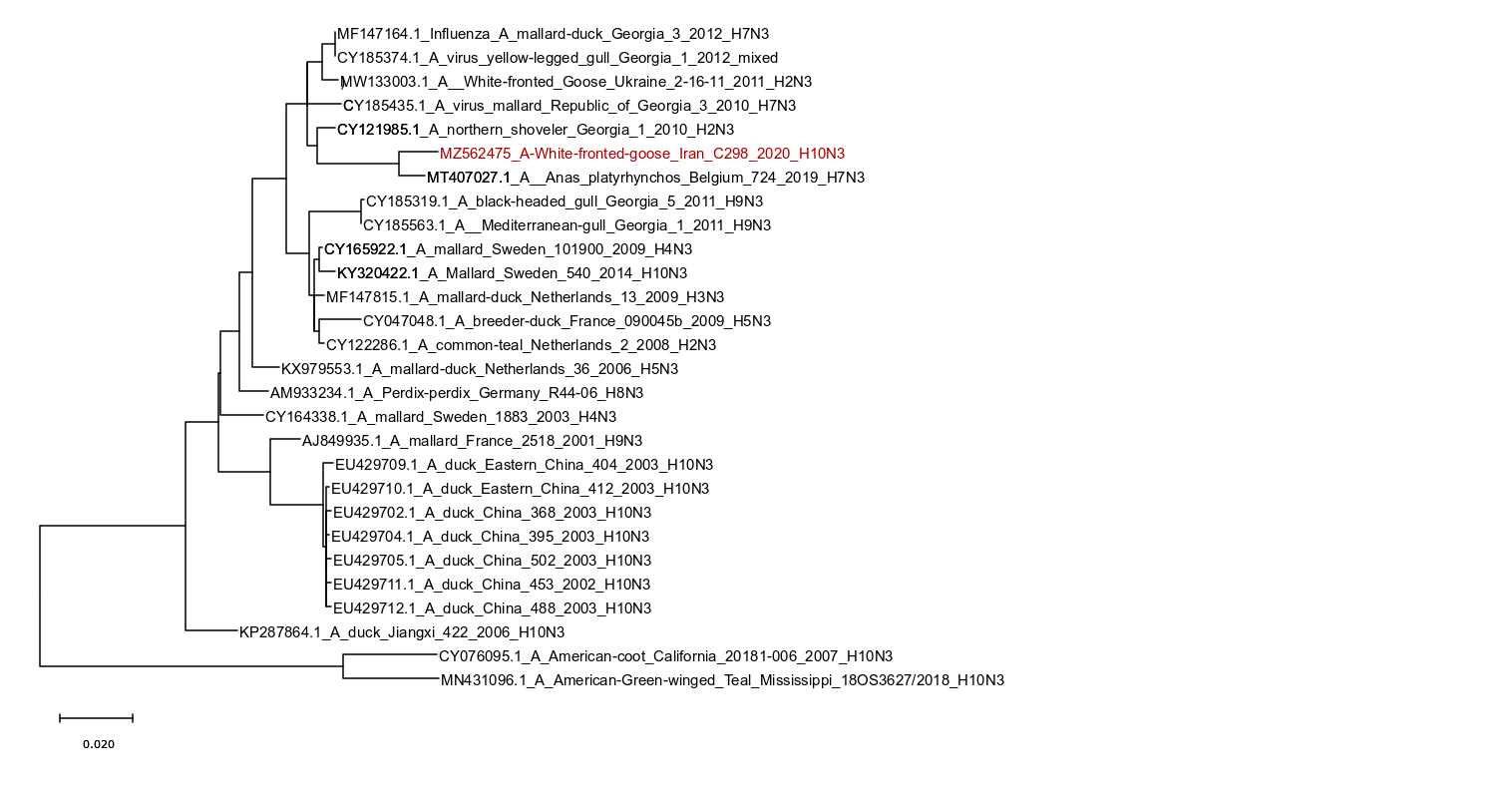


Supplemental figure 1J-K. HA H10 (upper panel) and NA, subtype N3
